# (p)ppGpp and DksA play a crucial role in reducing the efficacy of β-lactam antibiotics by modulating bacterial membrane permeability

**DOI:** 10.1128/spectrum.01169-24

**Published:** 2025-02-24

**Authors:** Meenal Chawla, Jyoti Verma, Shashi Kumari, Tushar Matta, Tarosi Senapati, Prabhakar Babele, Yashwant Kumar, Rupak K. Bhadra, Bhabatosh Das

**Affiliations:** 1 Functional Genomics Laboratory, Centre for Microbial Research, Translational Health Science and Technology Institute, Faridabad, India; 2 Non-communicable Diseases Division, Translational Health Science and Technology Institute 145787 , Faridabad, India; 3 Infectious Diseases and Immunology Division, CSIR-Indian Institute of Chemical Biology, Kolkata, India; The University of Texas at Tyler, Tyler, Texas, USA

**Keywords:** *Vibrio cholerae*, stringent response, (p)ppGpp, DksA, antibiotics, metabolome, proteome

## Abstract

The key signaling molecules in the bacterial stress-sensing pathway, the alarmone (p)ppGpp and the transcription factor DksA, play a crucial role in bacterial survival during nutritional deprivation and exposure to xenobiotics by modulating cellular metabolic pathways. In *Vibrio cholerae*, (p)ppGpp metabolism is solely linked with the functions of three proteins: RelA, SpoT, and RelV. The effects of threshold or elevated concentrations of (p)ppGpp on cellular metabolites and proteins, both in the presence and absence of DksA, have not yet been comprehensively studied in *V. cholerae* or other bacteria. We engineered the genome of *V. cholerae* to develop DksA null mutants in the presence and absence of (p)ppGpp biosynthetic enzymes. We observed that the N16:Δ*relA*Δ*relV*Δ*spoT*Δ*dksA V. cholerae* mutant, which lacks both (p)ppGpp and DksA, exhibits higher sensitivity to different ꞵ-lactam antibiotics compared with the wild-type (WT) strain. Our whole-cell metabolomic and proteome analysis revealed that the cell membrane and peptidoglycan biosynthesis pathways are significantly altered in the N16:Δ*relA*Δ*relV*Δ*spoT*, N16:Δ*dksA*, and N16:Δ*relA*Δ*relV*Δ*spoT*Δ*dksA V. cholerae* strains. Furthermore, the mutant strains displayed enhanced inner and outer membrane permeabilities in comparison to the WT strains. These results correlate with *V. cholerae’s* tolerance and survival against β-lactam antibiotics and may inform the development of adjuvants that inhibit stringent response modulators.

**IMPORTANCE:**

The (p)ppGpp biosynthetic pathway is widely conserved in bacteria. Intracellular levels of (p)ppGpp and the transcription factor DksA play crucial roles in bacterial multiplication and viability in the presence of antibiotics and/or other xenobiotics. The present findings have shown that (p)ppGpp and DksA significantly reduce the efficacy of ꞵ-lactam and other antibiotics by modulating the availability of peptidoglycan and cell membrane-associated metabolites by reducing membrane permeability. Nevertheless, the whole-cell proteome analysis of N16:Δ*relA*Δ*relV*Δ*spoT*, N16:Δ*dksA*, and N16:Δ*relA*Δ*relV*Δ*spoT*Δ*dksA* strains identified the biosynthetic pathways and associated enzymes that are directly modulated by the stringent response effector molecules. Thus, the (p)ppGpp metabolic pathways and DksA could be a potential target for increasing the efficacy of antibiotics and developing antibiotic adjuvants.

## INTRODUCTION

The stringent response (SR) is a key bacterial adaptation to stress caused by nutrient scarcity, allowing bacteria to rapidly adjust to changes in their environment. SR is characterized by major cellular reprogramming, which includes the downregulation of stable RNA (rRNA and tRNA) synthesis, upregulation of amino acid biosynthesis, and other central metabolism crucial for survival (1–4). SR is mediated by two small intracellular signaling molecules, guanosine 3’-diphosphate 5’-triphosphate (pppGpp) and guanosine 3’, 5’-(bis) diphosphates (ppGpp), collectively known as (p)ppGpp (5, 6). The (p)ppGpp is an authoritative regulator of nearly every facet of bacterial physiology, such as growth rate regulation, phase shift, toxin generation, biofilm development, motility, a plethora of additional virulence connections, and antimicrobial resistance (1, 2, 7). The non-DNA binding transcriptional factor DksA is also a SR master regulator (8). DksA was discovered as a multicopy suppressor of a *dnaK* mutant (9) and was later postulated to work at the transcriptional stage, where it was discovered to adhere to RNA polymerase (RNAP) (10, 11).

*Vibrio cholerae,* a well-studied human pathogen and the etiological agent of acute diarrheal disease cholera, is equipped with distinct (p)ppGpp metabolic pathways compared with other Gram-negative bacteria, including the model organism *Escherichia coli* (12). In *V. cholerae*, the regulation of intracellular (p)ppGpp metabolism involves two multidomain proteins, RelA and SpoT, along with a small alarmone synthetase known as RelV. The RelA and RelV enzymes exclusively synthesize (p)ppGpp, whereas SpoT is a bifunctional enzyme that possesses both (p)ppGpp hydrolase activity and a weak (p)ppGpp synthesizing ability (12–14). The (p)ppGpp alone or in combination with DksA interacts with RNAP to regulate transcriptional responses of more than 750 genes in *E. coli* (15, 16). The role of (p)ppGpp and DksA in modulating antimicrobial susceptibility has been studied in different pathogens (17–22); however, the mechanisms remain unclear.

In this study, we have done extensive genetic engineering on *V. cholerae*, aiming to investigate the impact of the (p)ppGpp and DksA deficits on modulating antimicrobial susceptibility. To elucidate the connection between these factors, *V. cholerae* strains with different genetic backgrounds, the N16:Δ*relA*Δ*relV*Δ*spoT* strain, which is unable to produce alarmone (p)ppGpp; the N16:Δ*dksA* strain, which lacks DksA; and the N16:Δ*relA*Δ*relV*Δ*spoT*Δ*dksA* strain, which is deficient in both (p)ppGpp and DksA, were used for antimicrobial susceptibility testing against different antibacterial agents commonly used to manage different infections. To gain insights into the underlying mechanisms, non-targeted metabolomics and proteomics approaches are employed to decode the metabolites and proteins that are directly connected with the stringent response. This integrated analysis aimed to unravel the changes associated with the observed differences in antibiotic susceptibility, shedding light on the role of (p)ppGpp and DksA in the adaptive responses of *V. cholerae* to antimicrobial exposure.

## RESULTS

### (p)ppGpp and DksA reduce the efficacy of antibiotics

**Fig 1:**
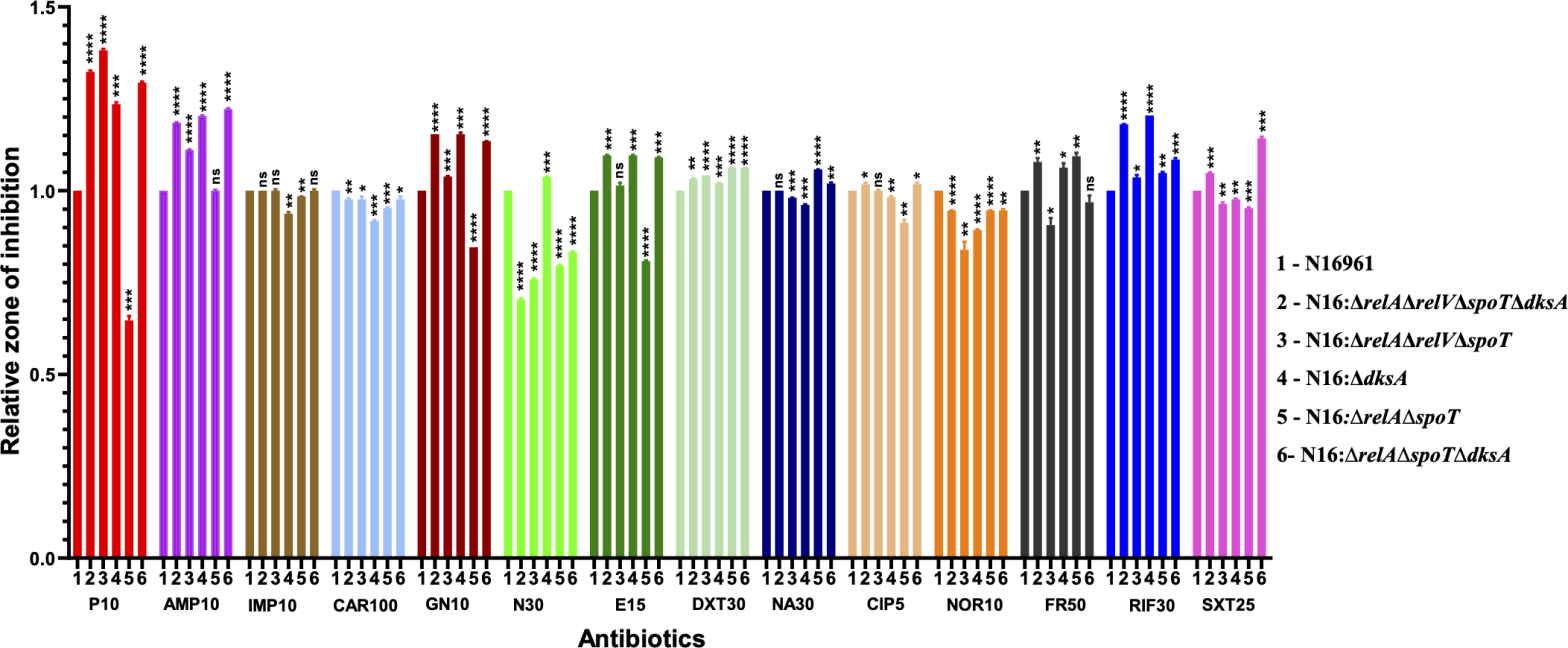
The graph represents the relative zone of inhibition of *V. cholerae* mutant strains, N16:Δ*relA*Δ*relV*Δ*spoT*Δ*dksA*, N16:Δ*relA*Δ*relV*Δ*spoT*, N16:Δ*dksA*, N16:∆*relA*∆*spoT,* and N16:∆*relA*∆*spoT*∆*dksA*, with different antibiotics, relative to N16961 strain. Unpaired *t*-test with Welch’s correction was used to determine statistical significance (*P* < 0.05), with significance calculated relative to the N16961 strain (*P*-values < 0.05 (*), <0.01 (**), *P*-values < 0.001 (***) and *P*-values < 0.0001 (****)). P10, Penicillin(10 µg); AMP10, Ampicillin(10 µg); IMP10, Imipenem(10 µg); CAR100, Carbenicillin(100 µg); GN10, Gentamicin(10 µg); N30, Neomycin(30 µg); E15, Erythromycin(15 µg); DXT30, Doxycycline(30 µg); NA30, Nalidixic acid(30 µg); CIP5, Ciprofloxacin(5 µg); NOR10, Norfloxacin(10 µg); FR50, Furazolidone(50 µg); RIF30, Rifampicin(30 µg); and SXT25, Trimethoprim/sulfamethoxazole(25 µg).

Antibiotic susceptibility tests by the disc diffusion method were conducted to measure the zone of clearance with different antibiotics in all the genetically modified strains (Table 1). A total of 14 antibiotics belonging to different classes were used. The absence of (p)ppGpp and loss of *dksA* functions is associated with bigger zones of clearance compared with wild-type (WT) strain for several antibiotics, indicating higher susceptibility (Fig. 1; Fig. S1). These results were also validated by determining the minimum inhibitory concentrations (MIC) of the mutant strains for all 14 antibiotics (Table 2; Table S1). Among the evaluated antibiotics, the N16:Δ*relA*Δ*relV*Δ*spoT*Δ*dksA* strain, a (p)ppGpp^0^Δ*dksA* mutant, exhibited the highest sensitivity compared with the WT strain for six antibiotics: penicillin, ampicillin, erythromycin, doxycycline, rifampicin, and sulfamethoxazole in both disc diffusion and MIC test. The strain N16:∆*relA*∆*spoT*, which has elevated levels of (p)ppGpp due to the presence of the (p)ppGpp synthetase enzyme RelV and a mutation in the hydrolase enzyme SpoT (12), shows significantly smaller zone diameters and similar or higher MICs for the β-lactam antibiotics ampicillin and penicillin compared with the WT strain. The absence of *dksA* gene has especially shown increased sensitivity to erythromycin, a macrolide antibiotic, as observed by both disc diffusion and MIC test (Fig. 1). DksA complementated strains of N16:Δ*relA*Δ*relV*Δ*spoT*Δ*dksA* and N16:Δ*dksA* was also evaluated through MIC testing. Although the complemented strains were resistant to ampicillin due to the inclusion of a resistance cassette during their construction, the N16:Δ*dksA-dksA* strain showed erythromycin and rifampicin MICs similar to those of the WT strain (Table S2). There is an intrinsic resistance to colistin and polymyxin B in all the strains, and resistance to kanamycin, spectinomycin, chloramphenicol, and zeocin in specific strains was due to the presence of resistance genes inserted during the genome engineering for gene knockout. These antibiotics were not considered for differential sensitivity tests.

**TABLE 1:** Relevant genotype and phenotype of wild-type and genetically modified *V. cholerae* strains used in the study

Strain	Genotype and phenotype	References
N16961	Wild-type, O1 El Tor, Stp^r^	(23)
NR13	N16961∆*relA,* Stp^r^, Kan^r^	(13)
BS1.1	N16961∆*relA*∆*spoT,* Stp^r^, Kan^r^, Cam^r^	(13)
NRV1	N16961∆*relV,* Stp^r^, Spec^r^	(12)
RRV1	N16961∆*relV*∆*relA,* Stp^r^, Spec^r^, Kan^r^	(12)
BRV1	N16961∆*relV*∆*relA*∆*spoT,* Stp^r^, Spec^r^, Kan^r^, Cam^r^	(12)
JV7	N16961Δ*dksA,* Stp^r^, Zeo^r^	This study
JV8	N16961∆*relA*∆*spoT*Δ*dksA,* Stp^r^, Kan^r^, Cam^r^, Zeo^r^	This study
JV9	N16961∆*relV*∆*relA*∆*spoT*Δ*dksA,* Stp^r^,Cam^r^,Spec^r^, Kan^r^, Zeo^r^	This study
MC1	N16961∆*relA*∆*dksA*, Strp^r^, Kan^r^, Zeo^r^	This study
MC3	N16961∆*relV*∆*relA*∆*dksA*, Stp^r^, Spec^r^, Kan^r^, Zeo^r^	This study
MC4	N16961∆*relV*∆*dksA,* Stp^r^, Spec^r^, Zeo^r^	This study
MC5	N16961Δ*dksA*::pBD62-DksA, Stp^r^, Zeo^r^, Amp^r^	This study
MC6	N16961∆*relV*∆*relA*∆*spoT*Δ*dksA*::pBD62-DksA*,* Stp^r^, Cam^r^, Spec^r^, Kan^r^, Zeo^r^, Amp^r^	This study

**TABLE 2:** Minimum Inhibitory concentration (MIC) of 14 different antibiotics for mutant strains

Antibiotics	N16961	N16:∆*dksA*	N16:∆*relA* ∆*relV*∆*spoT*	N16:∆*relA*∆*relV*∆*spoT*∆*dksA*	N16:∆*relA* ∆*spoT*	N16:∆*relA* ∆*spoT*∆*dksA*
Penicillin	5 µg/mL	2.5 µg/mL	2.5 µg/mL	2.5 µg/mL	10 µg/mL	2.5 µg/mL
Ampicillin	20 µg/mL	10 µg/mL	20 µg/mL	10 µg/mL	20 µg/mL	10 µg/mL
Imipenem	5 µg/mL	5 µg/mL	5 µg/mL	5 µg/mL	5 µg/mL	5 µg/mL
Carbenicillin	2.5 µg/mL	2.5 µg/mL	2.5 µg/mL	2.5 µg/mL	2.5 µg/mL	2.5 µg/mL
Gentamicin	5 µg/mL	5 µg/mL	5 µg/mL	5 µg/mL	5 µg/mL	5 µg/mL
Neomycin	20 µg/mL	20 µg/mL	>40 µg/mL	>40 µg/mL	>40 µg/mL	>40 µg/mL
Erythromycin	2.5 µg/mL	1.25 µg/mL	2.5 µg/mL	1.25 µg/mL	2.5 µg/mL	1.25 µg/mL
Doxycycline	100 ng/mL	100 ng/mL	100 ng/mL	25 ng/mL	100 ng/mL	50 ng/mL
Nalidixic acid	0.3125 µg/mL	0.3125 µg/mL	0.625 µg/mL	0.625 µg/mL	0.3125 µg/mL	0.3125 µg/mL
Ciprofloxacin	6.25 ng/mL	12.5 ng/mL	6.25 ng/mL	6.25 ng/mL	12.5 ng/mL	6.25 ng/mL
Norfloxacin	15.63 ng/mL	31.25 ng/mL	31.25 ng/mL	15.63 ng/mL	15.63 ng/mL	15.63 ng/mL
Furazolidone	0.625 µg/mL	0.625 µg/mL	0.625 µg/mL	0.625 µg/mL	0.625 µg/mL	0.625 µg/mL
Rifampicin	50 ng/mL	25 ng/mL	50 ng/mL	12.5 ng/mL	50 ng/mL	25 ng/mL
Sulfamethoxazole	20 µg/mL	5 µg/mL	40 µg/mL	5 µg/mL	20 µg/mL	20 µg/mL

### (p)ppGpp and DksA augment growth and multiplication of *V. cholerae* in the presence of a sub-lethal dose of the cell-wall-acting antibiotics

**Fig 2:**
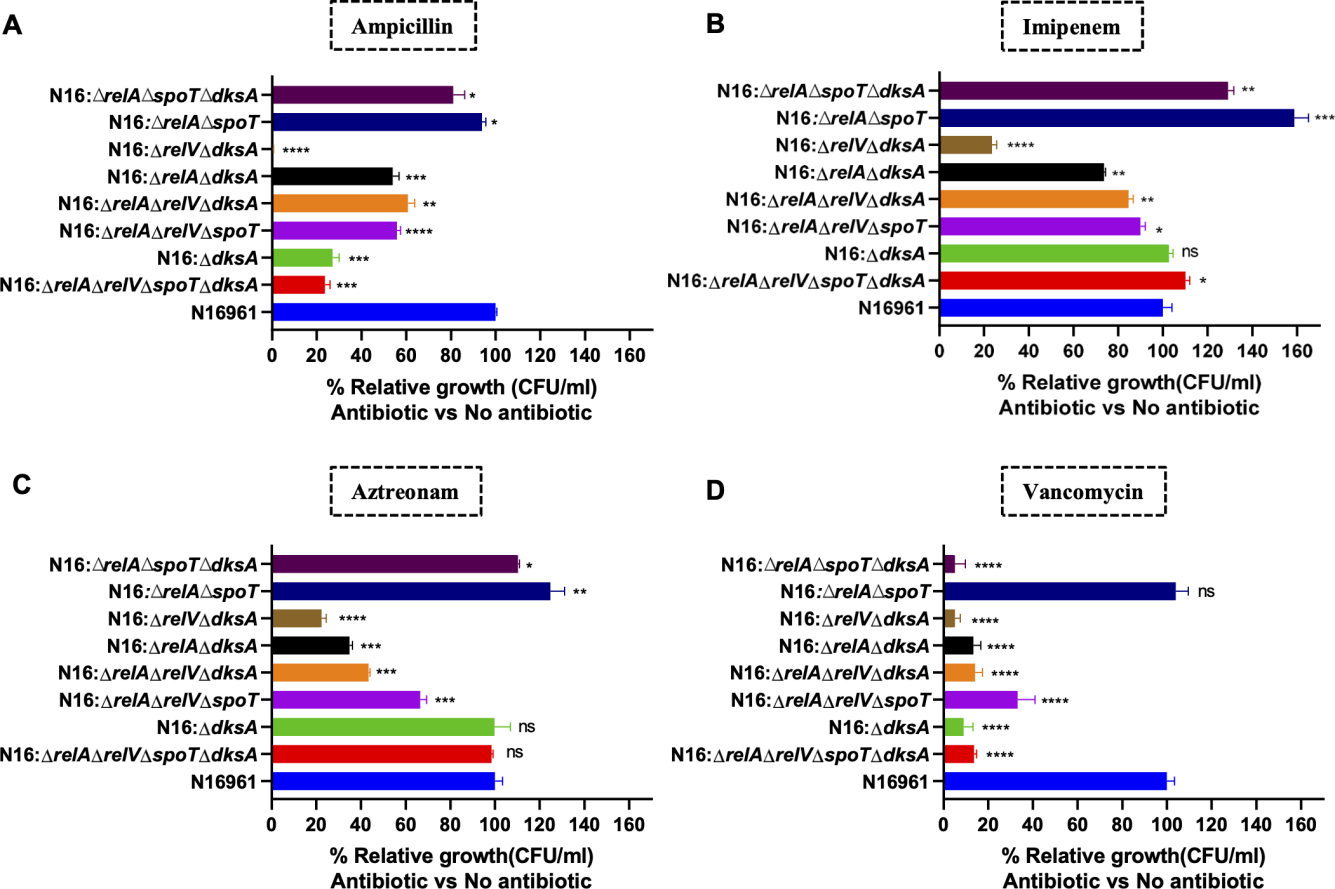
The graph showing the ability of growth of different mutant strains of *V. cholerae* in the presence of sublethal doses of (A) ampicillin, (**B**) imipenem, (**C**) aztreonam, and (D) vancomycin antibiotics. Unpaired *t*-test with Welch’s correction was used to determine statistical significance (*P* < 0.05), with significance calculated relative to the N16961 strain (*P*-values < 0.05 (*), <0.01 (**), *P*-values < 0.001 (***) and *P*-values < 0.0001 (****).

The increased sensitivity of (p)ppGpp and *dksA* mutant strains to β-lactam antibiotics prompted us to investigate the roles of these factors in the growth of bacteria when exposed to ampicillin, aztreonam, imipenem, and vancomycin antibiotics in the growth medium. To comprehend their significance, we selected these four antibiotics that primarily act on bacterial cell wall biosynthesis and assessed their activity in DksA single mutant of N16961 as well as the DksA mutant with mutations in the (p)ppGpp synthetase enzymes RelV and RelA, and the (p)ppGpp hydrolase enzyme SpoT. Additionally, the strain with (p)ppGpp null (N16:Δ*relA*Δ*relV*Δ*spoT*Δ*dksA*), where all three (p)ppGpp metabolism enzymes were mutated along with DksA, was also assessed. All these strains were selected for understanding the roles DksA and the individual genes involved in (p)ppGpp metabolism in relation to antibiotic resistance. Of four antibiotics, two antibiotics, ampicillin and vancomycin, exhibited a decrease in the growth of N16:Δ*relA*Δ*relV*Δ*spoT*Δ*dksA* and N16:Δ*dksA* strains compared with WT strain N16961. In contrast, the N16:∆*relA*∆*spoT* strain demonstrated higher growth to ampicillin and vancomycin compared with the N16:Δ*relA*Δ*relV*Δ*spoT*Δ*dksA* strain. However, when compared with the N16961 strain, N16:∆*relA*∆*spoT* shows lower growth for ampicillin, whereas growth for vancomycin is similar, with only a slight non-significant increase. For the N16:∆*relA*∆*spoT*∆*dksA* strain, growth is higher for ampicillin compared with the N16:Δ*relA*Δ*relV*Δ*spoT*Δ*dksA* strain but lower compared with the N16961 strain. For vancomycin, the N16:∆*relA*∆*spoT*∆*dksA* strain exhibits lower growth compared with both the N16:Δ*relA*Δ*relV*Δ*spoT*Δ*dksA* and N16961 strains (Fig. 2A and 2D). For imipenem and aztreonam, reduced growth was observed in the (p)ppGpp and *dksA* mutant strains, except for the N16:Δ*relA*Δ*relV*Δ*spoT*Δ*dksA* and N16:Δ*dksA* strains. This suggests that (p)ppGpp may play a more significant role in resistance to these two antibiotics. None of the bacterial strains had antibiotic resistance genes against these selected antibiotics. Thus, results suggest that the level of (p)ppGpp and presence and absence of DksA are directly correlated with the growth ability of bacterial cells. The (p)ppGpp and DksA contribute to baseline resistance for some of the tested antibiotics and thus help *V. cholerae* to sustain in the presence of these antimicrobial agents. Similarly, DksA, either independently or in conjunction with (p)ppGpp, can modulate transcriptional regulation of various genes involved in metabolic functions directly or indirectly linked to antibiotic resistance.

### Differential production of peptidoglycan and cell membrane-associated metabolites in (p)ppGpp^0^ and DksA mutants

**Fig 3:**
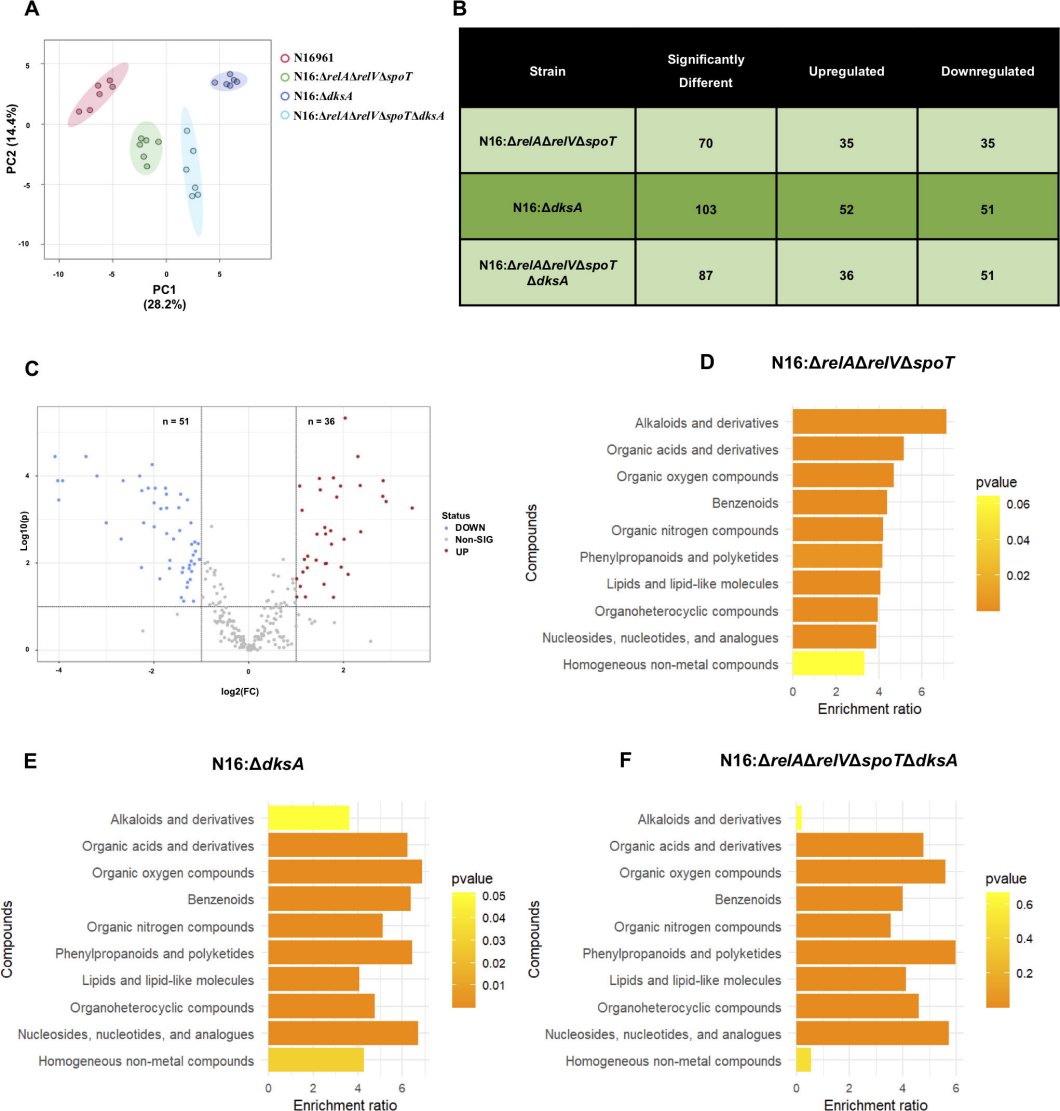
Chemometric analysis of metabolites among N16:Δ*relA*Δ*relV*Δ*spoT*, N16:Δ*dksA*, N16:Δ*relA*Δ*relV*Δ*spoT*Δ*dksA,* and N16961 controls. (**A**) Two-dimensional principal component analysis (PCA) score plot of four groups. (**B**) Table illustrating metabolites significantly upregulated and downregulated in N16:Δ*relA*Δ*relV*Δ*spoT*, N16:Δ*dksA*, and N16:Δ*relA*Δ*relV*Δ*spoT*Δ*dksA* strains. A fold-change threshold of ±2 was applied, and statistical significance was determined using unpaired *t*-tests with FDR adjustment at *P* = 0.05. (**C**) Volcano plot of the 291 identified metabolites of N16:Δ*relA*Δ*relV*Δ*spoT*Δ*dksA* by LC–MS. The volcano plot shows the fold-change (x-axis) versus the significance (y-axis) of the 291 metabolites. The vertical and horizontal dotted lines show the cutoff of fold-change =  ± 2, and of FDR-adjusted *P*-value  =  0.05, respectively. Metaboanalyst-based chemical-enrichment analysis of the identified metabolites in (D) N16:Δ*relA*Δ*relV*Δ*spoT*, (**E**) N16:Δ*dksA,* and (F) N16:Δ*relA*Δ*relV*Δ*spoT*Δ*dksA* strains.

To unravel the mechanisms contributing to the heightened susceptibility of mutant strains to β-lactam antibiotics, an untargeted metabolomic analysis was conducted. Liquid chromatography coupled mass spectrometry-based profiling was used to study the diversity and abundance of metabolites of genetically engineered *V. cholerae* strains with or without (p)ppGpp synthetase/hydrolase encoding genes and their combination with the presence and absence of DksA functions. This comparison is the first metabolomic study to determine the chemical fingerprint of *V. cholerae* (p)ppGpp null and DksA variant strains, as well as the important biochemical pathways involved in the formation of essential metabolites in WT and (p)ppGpp and DksA mutant *s*trains. A total of around 291 metabolites were identified. The combined and individual effects of (p)ppGpp levels and the DksA mutation have been studied, and each mutant strain showed a substantial difference from the WT strain. The PCA plot illustrates the differences between the N16961, N16:Δ*relA*Δ*relV*Δ*spoT*, N16:Δ*dksA*, and N16:Δ*relA*Δ*relV*Δ*spoT*Δ*dksA* strains (Fig. 3A). The significantly upregulated and downregulated metabolites for these mutant strains compared with N16961, with a fold-change threshold of ±2 is illustrated in Fig. 3B. The significance versus fold-change of all the identified metabolites for the N16:Δ*relA*Δ*relV*Δ*spoT*Δ*dksA* strain is shown in the volcano plot (Fig. 3C) and for N16:Δ*relA*Δ*relV*Δ*spoT* and N16:Δ*dksA* in Fig. S2. Chemical enrichment analysis was performed in order to highlight the chemical classes significantly altered in N16:Δ*relA*Δ*relV*Δ*spoT*, N16:Δ*dksA*, and N16:Δ*relA*Δ*relV*Δ*spoT*Δ*dksA* strains with respect to N16961 strain individually. The chemical enrichment analysis further corroborated the differential results in these mutant strains (Fig. 3D through F, respectively). Chemical classes with a high enrichment ratio for N16:Δ*relA*Δ*relV*Δ*spoT* strain include alkaloids, organic acids, and lipids among others, and chemical classes with a high enrichment ratio for N16:Δ*dksA* strain include organic oxygen compounds, nucleosides, nucleotides, phenylpropanoids, and polyketides. However, for N16:Δ*relA*Δ*relV*Δ*spoT*Δ*dksA* strain, phenylpropanoids, polyketides, nucleosides, and nucleotides chemical classes had a high enrichment ratio.

**Fig 4:**
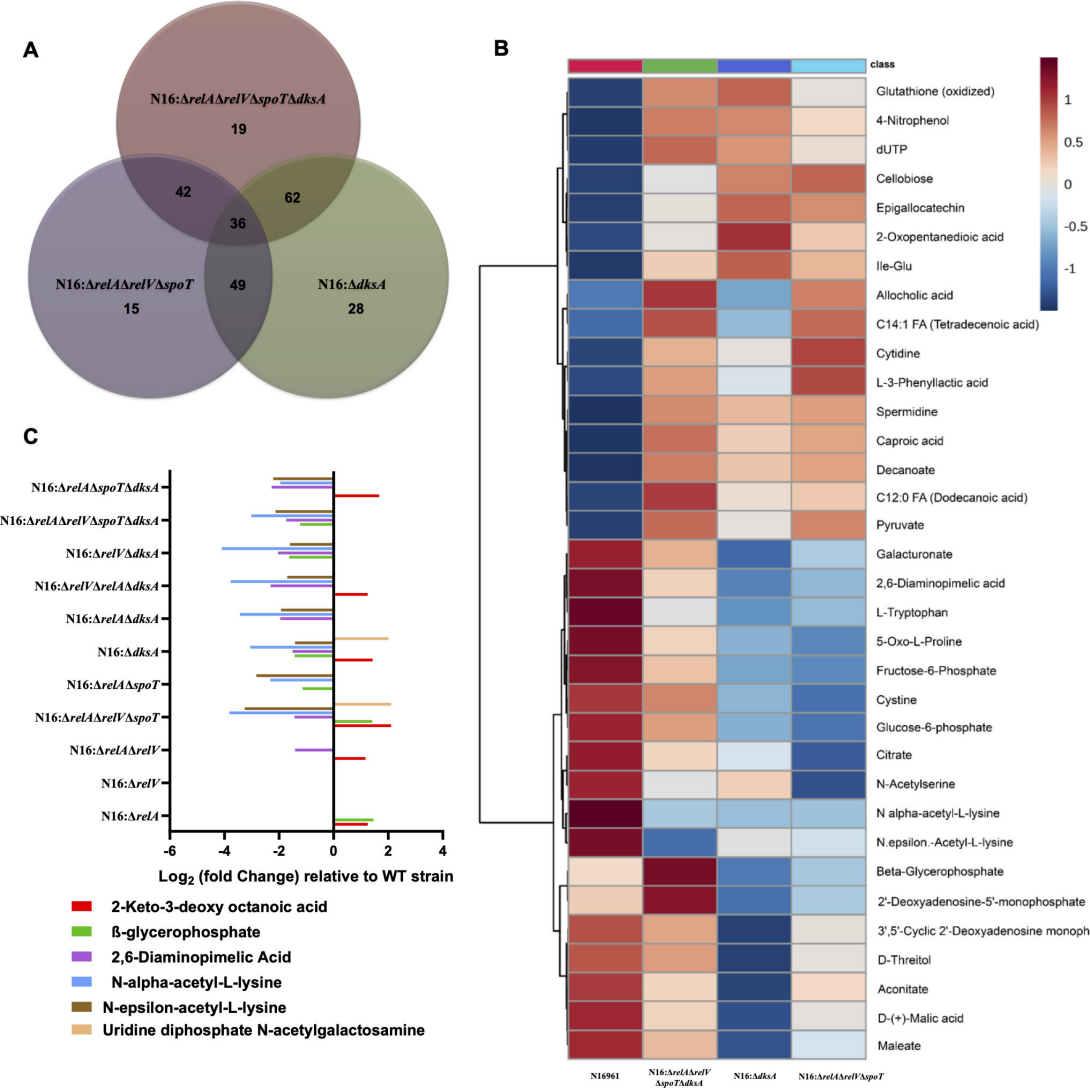
(A) Venn diagram representing the numbers of altered metabolites in the N16:Δ*relA*Δ*relV*Δ*spoT*, N16:Δ*dksA,* and N16:Δ*relA*Δ*relV*Δ*spoT*Δ*dksA* mutant strains. Thirty-six differential metabolites are overlapped among the three strains. (**B**) Heatmap analysis of 36 overlapped metabolites among the N16:Δ*relA*Δ*relV*Δ*spoT*, N16:Δ*dksA,* and N16:Δ*relA*Δ*relV*Δ*spoT*Δ*dksA* strains. (**C**) Log_2_ fold-change of six important cell wall and membrane differentiated metabolites in the mutant strains compared with the WT (N16961) strain.

We identified differential patterns in metabolites associated with amino acid metabolism, nucleotide metabolism, and the tricarboxylic acid (TCA) cycle in (p)ppGpp and DksA mutant strains. In addition to elucidating their roles in these key cellular processes, our study unveiled regulatory effects by (p)ppGpp and DksA on metabolites specifically related to cell walls and membrane components. The nucleotide sugar uridine diphosphate N-acetylgalactosamine, also known as UDP-GalNAc, is the precursor for the formation of lipid A of lipopolysaccharide (LPS), which constitutes the majority of Gram-negative bacterial outer membrane (24). The levels of UDP-GalNAc in N16:Δ*relA*Δ*relV*Δ*spoT* strain were observed to be upregulated; in contrast, no change in UDP-GalNAc levels was observed in N16:∆*relA*∆*spoT* strain (Fig. 4C). The other crucial component of LPS, 2-Keto-3-deoxy octanoic acid (KDO) present in the outermost leaflet of the Gram-negative bacterial outer membrane, was observed to be higher in the N16:Δ*relA*Δ*relV*Δ*spoT* and N16:Δ*dksA* strain compared with WT strain (Fig. 4C). Lipid IVA receives the two Kdo sugar from the core oligosaccharide. The enzyme WaaA, formerly known as KdtA, mediates this step by successively adding Kdo groups to the lipid IVA from activated Kdo (CMP-Kdo) (25–28).

Subsequently, several metabolites were observed to be regulated by both (p)ppGpp alarmone and DksA transcription factor, either through collaborative or antagonistic mechanisms. The metabolites of N16:Δ*relA*Δ*relV*Δ*spoT*, N16:Δ*dksA*, and N16:Δ*relA*Δ*relV*Δ*spoT*Δ*dksA* strains are depicted in Venn diagram (Fig. 4A). A total of 36 significantly altered metabolites were discovered to be shared among these three mutant strains of *V. cholerae,* and these metabolites were closely investigated. Heatmap analysis of 36 overlapped metabolites is shown in Fig. 4B. Among the shared metabolites, few cell wall- and membrane-specific metabolites were observed to be differently regulated, and glycerol-2-phosphate (also known as β-glycerophosphate), was involved in the synthesis of cell membrane. In contrast to the N16:Δ*relA*Δ*relV*Δ*spoT* strain, β-glycerophosphate was shown to be downregulated in N16:Δ*dksA* and N16:Δ*relA*Δ*relV*Δ*spoT*Δ*dksA* strains, illustrating antagonistic regulation. However, in N16:∆*relA*∆*spoT* strain, β-glycerophosphate was observed to be downregulated, and there was no change in the level of β-glycerophosphate in the N16:∆*relA*∆*spoT*∆*dksA* strain (Fig. 4C). Apart from this, 2,6-diaminopimelic acid (DAP), a major metabolite that is frequently found in the peptide links of NAM-NAG chains that constitute the cell wall of Gram-negative bacteria was shown to be drastically downregulated in N16:Δ*relA*Δ*relV*Δ*spoT*, N16:Δ*dksA*, and N16:Δ*relA*Δ*relV*Δ*spoT*Δ*dksA* strains (Fig. 4C). Bacteria with a DAP shortage nevertheless grow normally but are not able to produce new peptidoglycan for their cell walls (29, 30). Also, N-α-acetyl-L-lysine and N-ε-acetyl-L-lysine, which is necessary for bacteria to produce lysine for protein synthesis, and cell wall biosynthesis, were similarly downregulated in all mutant strains (Fig. 4C). The formation and integrity of cell wall and membrane could be compromised, suggesting a membrane re-composition linked to the basal levels of (p)ppGpp and presence and absence of DksA.

### Whole-cell proteome of (p)ppGpp and DksA mutant strains showed a reduced level of cell-wall biosynthetic enzymes

**Fig 5:**
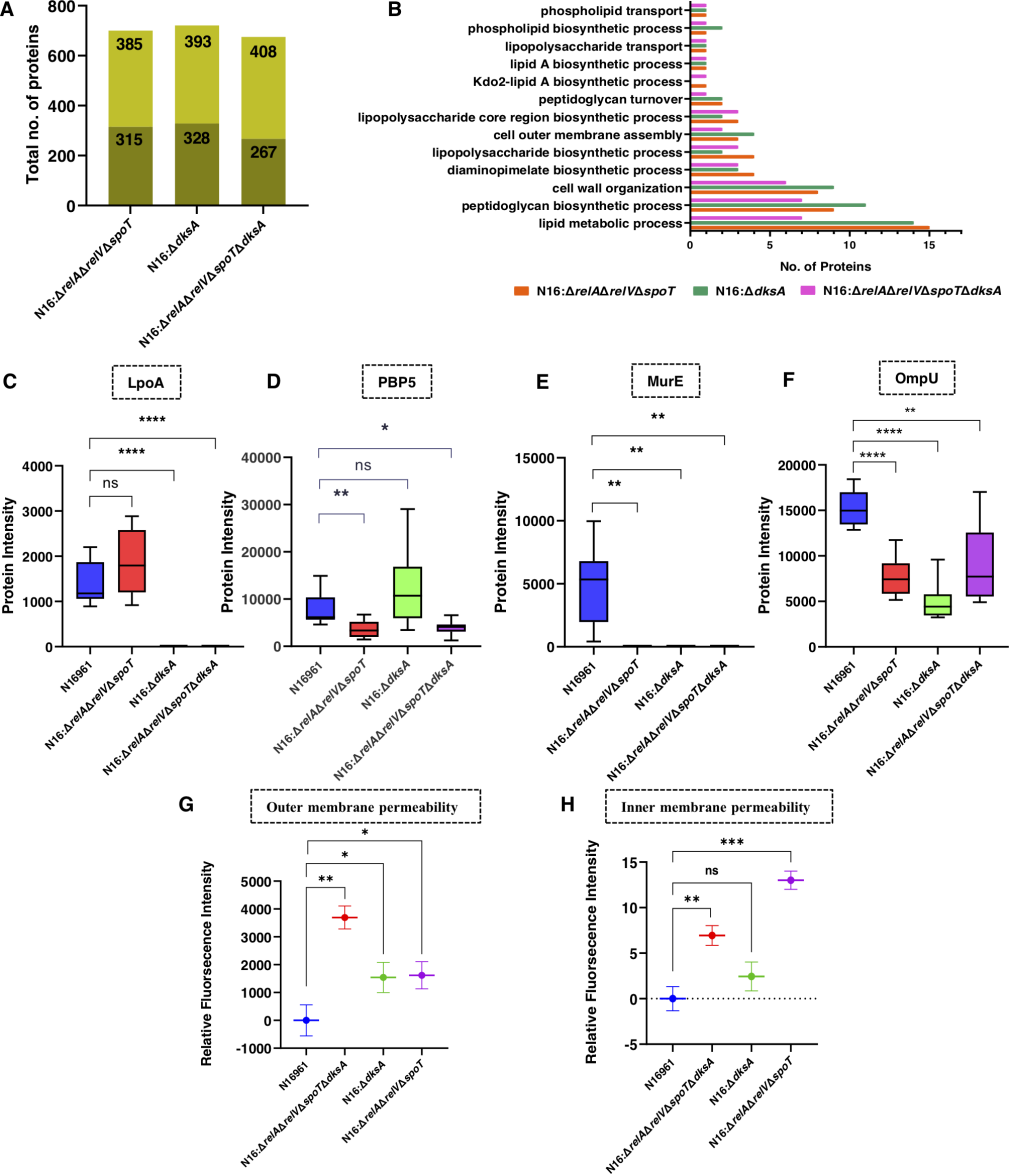
(A) Total number of proteins identified by qualitative (lighter region) and quantitative analysis (darker region with fold-change ≥1.5 wrt WT) in N16:Δ*relA*Δ*relV*Δ*spoT*, N16:Δ*dksA,* and N16:Δ*relA*Δ*relV*Δ*spoT*Δ*dksA* strains. (**B**) Comparison of cell wall and membrane-associated pathways of differentially regulated proteins (mutant vs. WT) in N16:Δ*relA*Δ*relV*Δ*spoT*, N16:Δ*dksA,* and N16:Δ*relA*Δ*relV*Δ*spoT*Δ*dksA* strains. (**C-F**) Box plot of protein intensities (mean area under the curve) for N16961, N16:Δ*relA*Δ*relV*Δ*spoT*, N16:Δ*dksA,* and N16:Δ*relA*Δ*relV*Δ*spoT*Δ*dksA* strains. Each plot represents a different protein. (C- LpoA, D- PBP5, E- MurE, F- OmpU). (G-H) Relative fluorescence intensity for outer and inner membrane permeability assays, detected by NPN and PI uptake, respectively, in mutant strains compared with the WT strain [Welch’s *t*-test, (*P* < 0.05)]

The regulatory roles of (p)ppGpp and DksA in influencing various cell wall-associated biochemical pathways have been elucidated through metabolomic analyses. To enhance the mechanistic understanding of these regulatory processes, we explored the whole-cell proteome of mutant strains N16:Δ*relA*Δ*relV*Δ*spoT*, N16:Δ*dksA*, and N16:Δ*relA*Δ*relV*Δ*spoT*Δ*dksA*. Utilizing the SWATH-MS platform, a total of 707, 731, and 684 proteins were quantified in N16:Δ*relA*Δ*relV*Δ*spoT*, N16:Δ*dksA*, and N16:Δ*relA*Δ*relV*Δ*spoT*Δ*dksA* strains, respectively, in comparison to the N16961 strain (Fig. 5A). Several proteins involved in cell wall synthesis, organization, peptidoglycan biosynthesis, phospholipid transport, lipopolysaccharide transport, and diaminopimelate biosynthetic processes exhibited differential regulation in mutant strains (Fig. 5B).

The penicillin-binding protein activator LpoA (PBP activator LpoA) was qualitatively identified in the WT and N16:Δ*relA*Δ*relV*Δ*spoT* strains but remained undetected in N16:Δ*dksA* and N16:Δ*relA*Δ*relV*Δ*spoT*Δ*dksA* strains (Fig. 5C). Furthermore, serine-type D-Ala-D-Ala carboxypeptidase (PBP5), a penicillin-sensitive, membrane-bound enzyme associated with resistance to β-lactam antibiotics was observed to be downregulated in N16:Δ*relA*Δ*relV*Δ*spoT* (FC of 0.46) and N16:Δ*relA*Δ*relV*Δ*spoT*Δ*dksA* (FC of 0.512) strains (Fig. 5D). The enzymes involved in the DAP biosynthetic pathway exhibited distinct regulatory patterns in mutant strains. Notably, UDP-MurNAc-L-Ala-D-Glu:meso-diaminopimelate ligase (MurE), the enzyme responsible for integrating meso-diaminopimelic acid into the nucleotide precursor UDP-N-acetylmuramoy-L-alanyl-D-glutamate (UMAG), was qualitatively identified in the N16961 strain but remained undetected in N16:Δ*relA*Δ*relV*Δ*spoT*, N16:Δ*dksA*, and N16:Δ*relA*Δ*relV*Δ*spoT*Δ*dksA* strains (Fig. 5E). DAP decarboxylase, which catalyzes the final step of the DAP pathway, was also significantly downregulated in N16:Δ*relA*Δ*relV*Δ*spoT* (FC of 0.644) and N16:Δ*dksA* (FC of 0.482) strains (Fig. S3A). This alignment with metabolomics data provides additional layers of validation, reinforcing the impact of (p)ppGpp and DksA on cell wall synthesis and dynamics by regulating the DAP pathway. In addition to this, outer membrane protein U (OmpU) was observed to be downregulated in N16:Δ*relA*Δ*relV*Δ*spoT* (FC of 0.5), N16:Δ*dksA* (FC of 0.321), and N16:Δ*relA*Δ*relV*Δ*spoT*Δ*dksA* (FC of 0.585) strains (Fig. 5F). Real-time PCR analysis further confirmed a significant reduction in *ompU* expression in N16:Δ*relA*Δ*relV*Δ*spoT*Δ*dksA* strain. In contrast, the expression of *ompT* was observed to be higher in N16:Δ*relA*Δ*relV*Δ*spoT*Δ*dksA* compared with N16961 strain when treated with a sub-lethal concentration of antibiotic ampicillin (Fig. S3B). Other than cell wall and membrane-related proteins, we have also found numerous proteins showing differential expression in mutant strains in comparison to WT strains. These differentially expressed proteins predominantly belong to pathways involving metabolism, replication and translational regulation, and stress response. These findings strongly suggest the modulation of additional cellular pathways, which may be directly and/or indirectly linked with the increased susceptibility of antibiotics in (p)ppGpp and DksA mutant strains.

### Increased membrane permeability of N16:Δ*relA*Δ*relV*Δ*spoT* and N16:Δ*relA*Δ*relV*Δ*spoT*Δ*dksA V. cholerae* strains

Metabolomic and proteomic analyses uncovered compelling evidence suggesting that (p)ppGpp and DksA might affect bacterial cell membrane synthesis, which in turn could impact the structural and functional integrity of the cell membrane. Based on these findings, we have hypothesized that (p)ppGpp and DksA mutant strains may exhibit alterations in membrane permeability. To determine the effect of (p)ppGpp and DksA on inner membrane permeability (IMP) and outer membrane permeability (OMP), the assay using specific dyes was performed in N16:Δ*relA*Δ*relV*Δ*spoT*, N16:Δ*dksA*, and N16:Δ*relA*Δ*relV*Δ*spoT*Δ*dksA* strains. The OMP was evaluated by using NPN uptake assay. NPN is a neutral hydrophobic fluorescent probe, which is usually excluded by the OM but when it partitions into the OM, it demonstrates increased fluorescence intensity. NPN fluorescence was significantly enhanced to the same level in N16:Δ*relA*Δ*relV*Δ*spoT* and N16:Δ*dksA* strains, and a markedly enhanced effect was observed in N16:Δ*relA*Δ*relV*Δ*spoT*Δ*dksA*. The IMP was assessed by PI uptake assay as PI is a membrane-impermeant stain, only labels the bacteria having compromised IM. In contrast to OMP, the IMP was significantly enhanced in N16:Δ*relA*Δ*relV*Δ*spoT* and N16: Δ*relA*Δ*relV*Δ*spoT*Δ*dksA* strains; however, no significant difference was observed in N16:Δ*dksA* strain (Fig. 5G and H).

## DISCUSSION

Previous studies, including our own, have highlighted the critical roles of (p)ppGpp and DksA in the stringent response. These molecules are essential for microbial survival and adaptation under conditions of nutrient limitation and various environmental stresses (8, 12). DksA and (p)ppGpp direct cellular resources from growth and multiplication processes to survival-associated metabolic activities. However, intriguing disparities emerge at both the genotypic and phenotypic levels (17, 31). Stringent response modulators have been reported to modulate various cellular processes in Gram-negative bacteria such as fatty acid metabolism, amino acid metabolism, and flagellar synthesis, thereby determining virulence, pathogenicity, and bacterial survival under stressful conditions (32–35). The current study pursues to address the intricate interplay between (p)ppGpp and DksA in the modulation of antibiotic resistance in the absence of antibiotic resistance gene. The investigation spans phenotypic, metabolomic, and proteomic analyses in (p)ppGpp- and DksA-deficient strains, providing a comprehensive understanding of the potential regulatory mechanisms underlying the increased antibacterial susceptibility in these mutant bacterial strains.

At phenotypic levels, we have observed that (p)ppGpp- and DksA-deficient strains of *V. cholerae* N16:Δ*relA*Δ*relV*Δ*spoT*Δ*dksA* exhibit enhanced susceptibility to different antibiotics. Notably, the most pronounced effects were observed for β-lactam antibiotics. The growth assay with β-lactam antibiotic ampicillin, imipenem, and aztreonam further underscored the susceptibility of (p)ppGpp- and DksA-deficient strains. In the absence of the resistance genes and in the presence of sub-lethal concentrations of cell-wall-inhibiting antibiotics, growth of (p)ppGpp- and DksA-deficient strains was significantly diminished compared with WT strain and strain with higher levels of (p)ppGpp, except for the DksA-deficient strains in the presence of sub-lethal concentration of aztreonam. Our findings aligned with earlier studies conducted in *E. coli* that have highlighted that (p)ppGpp-deficient (Δ*rel*AΔ*spo*T) mutant was more susceptible to cell-wall acting and other different classes of antimicrobial agents than the WT (19). The *∆dksA* strain has also been demonstrated in *E. coli* to be more sensitive to antimicrobial drugs such as β-lactams, aminoglycosides, quinolones, and tetracyclines relative to the WT strain (20). This susceptibility pattern is also observed in *Acinetobacter baumannii,* where both (p)ppGpp-deficient and DksA-deficient strains exhibited increased susceptibility to antimicrobial agents (18, 22). Despite the conventional application for targeting Gram-positive bacteria, vancomycin has also been included in our investigation as it has been extensively studied for its impact on (p)ppGpp-deficient strains in bacteria such as *Enterococcus faecalis, Staphylococcus aureus, Enterococcus faecium,* and *Bacillus subtilis* (36–38). We observed that growth is markedly reduced in (p)ppGpp and DksA-deficient strains of *V. cholerae* in the presence of sub-lethal concentration of vancomycin.

The mass spectrometry-based profiling method was conducted to study the level of metabolites in genetically engineered *V. cholerae* strains. In our current investigation, we observed that 50% of the differentially regulated metabolites were downregulated in both N16:Δ*relA*Δ*relV*Δ*spoT* and N16:Δ*dksA* strains. Additionally, in the N16:Δ*relA*Δ*relV*Δ*spoT*Δ*dksA* strain, 58% of the differently regulated metabolites exhibited downregulation. This highlights a predominant trend of metabolic alterations associated with (p)ppGpp and DksA in *V. cholerae*. Within the realm of membrane-specific metabolites, distinct patterns emerged. The UDP-GalNAc and KDO, which are precursors for Kdo2-lipid A, a principle and essential component of the OM of Gram-negative bacteria, are observed to be upregulated in N16:Δ*relA*Δ*relV*Δ*spoT* strain. Glycerol-2-phosphate displayed an antagonistic regulation, with elevated levels in the N16:Δ*relA*Δ*relV*Δ*spoT* strain and decreased levels in both the N16:Δ*dksA* and N16:∆*relA*∆*spoT* strains. Furthermore, the absence of single or both SR modulators resulted in the downregulation of DAP levels. The previous transcriptional study conducted in *E. coli* has highlighted the substantial differences in the regulation of a significant number of genes involved in cell wall maintenance, plasma membrane function, and lipopolysaccharide (LPS) metabolism (15). Our current research aligns with prior findings, emphasizing that (p)ppGpp and DksA play a significant impact in shaping the dynamics of amino acid, nucleotide, and carbohydrate metabolism (6, 39).

To further confirm the findings of our phenotypic and metabolomics study, the total proteome of N16:Δ*relA*Δ*relV*Δ*spoT*, N16:Δ*dksA,* and N16:Δ*relA*Δ*relV*Δ*spoT*Δ*dksA* in comparison to N16961 strain was explored. The proteomic analysis revealed the differential regulation of various cell membranes and PG-associated proteins in the mutant strains. The N16:Δ*relA*Δ*relV*Δ*spoT*Δ*dksA* strain exhibits reduced expressions of LpoA, PBP5, and OmpU, suggesting alterations in cell wall integrity and permeability of the outer membrane synthesis. LpoA has been reported to be essential for transpeptidase function of penicillin-binding protein 1A (PBP1a) and also to activate the upstream step of peptidoglycan polymerization (40). This indicates that the transpeptidase function of PBP1a and peptidoglycan polymerization might be indirectly linked to the expression of (p)ppGpp in combination with DksA. In line with the findings, the study in *E. coli* has highlighted that deletion of PBP5 makes the bacteria 4-fold to 8-fold more susceptible to β-lactam antibiotics (41). Additionally, a recent study has highlighted the impact of (p)ppGpp on LPS biosynthesis by inhibiting LpxA, the first enzyme involved in LPS biosynthesis pathway (42). However, in our study, no significant influence on LpxA was observed. Furthermore, as highlighted by Grant et al. in a recent study, the OmpU mutant strain of N16961 exhibits greater susceptibility to a variety of antimicrobials compared with the WT strain (43). In our study, we observed reduced expression of OmpU and increased expression of OmpT when treated with a sub-lethal concentration of β-lactam antibiotic. Notably, OmpT and other porins play a key role in facilitating antibiotic influx. In this context, previous studies have shown that antibiotic flux in strains expressing only OmpT is higher than in those producing only OmpU, indicating that OmpT is more receptive than OmpU (44). The reliability of our metabolomics and proteomics findings is strengthened by the observed increased permeability of both IM and OM in the mutant strains.

**Fig 6:**
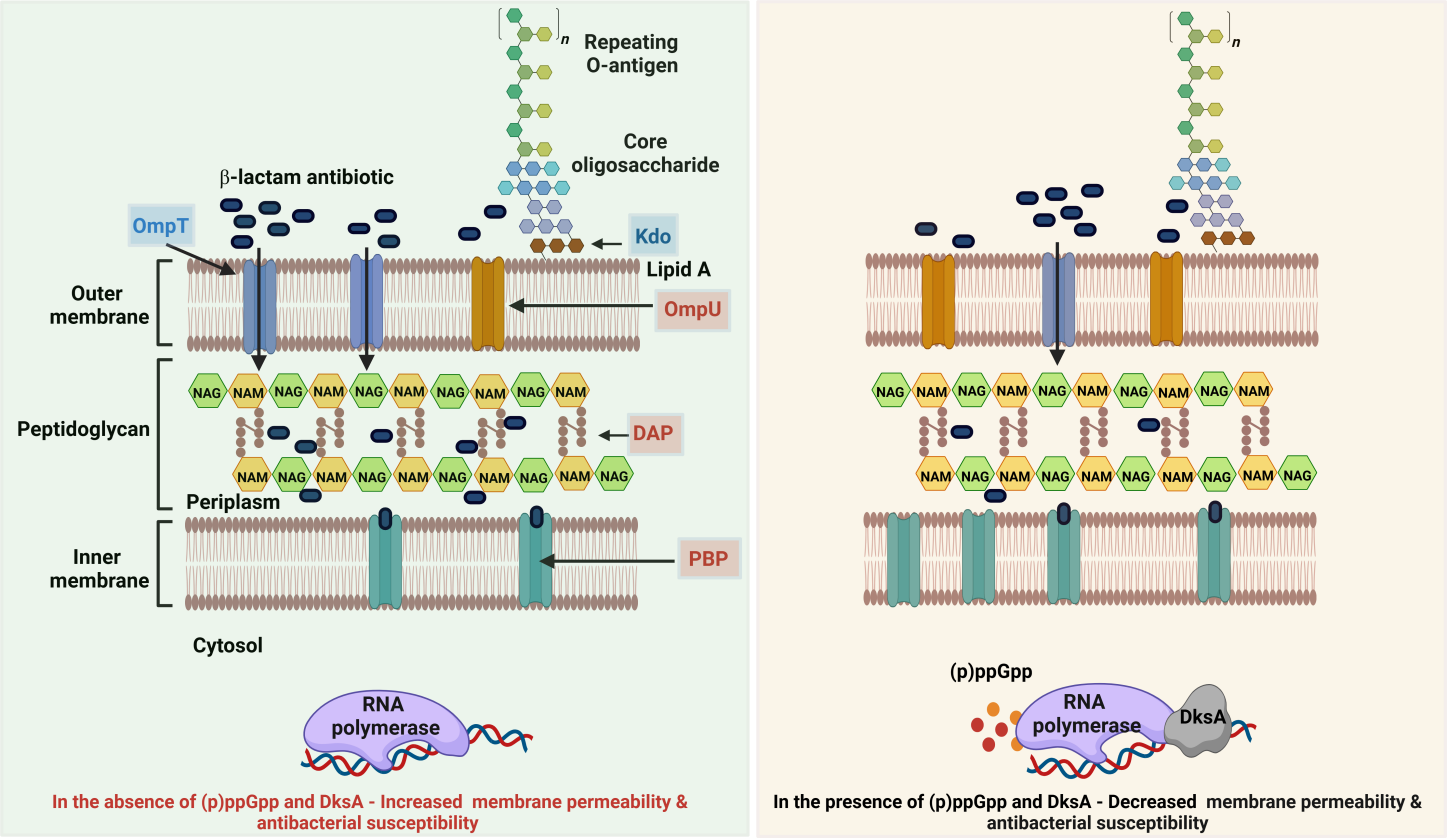
Schematic representation illustrating the heightened susceptibility to β-lactam antibiotics in (p)ppGpp and DksA mutant strain of *V. cholerae*. The increased susceptibility is attributed to distinct factors, including (i) decreased expression of penicillin-binding protein 5 (PBP5), (ii) decreased expression of outer membrane protein U (OmpU), (iii) increased expression of outer membrane protein T (OmpT), (iv) upregulation of 2-keto-3-deoxy octanoic acid (Kdo), and (v) downregulation of 2,6-diaminopimelic acid (DAP), indicating altered regulation of cell wall integrity and permeability of cell membrane in the mutant strains.

In summary, our integrated genetic, proteomics, and metabolomics approach unveils a distinct regulatory network orchestrated by (p)ppGpp and DksA, elucidating their influence on reduced susceptibility to β-lactam antibiotics. The proposed mechanism for the increased susceptibility of (p)ppGpp and DksA mutant strains of *V. cholerae* to cell-wall-inhibiting antibiotics, as highlighted in the study, is illustrated in Fig. 6. This mechanism involves several factors, including alterations in cell membrane and peptidoglycan synthesis activity, reduced expression of penicillin-binding proteins (PBPs), and differential expression of outer membrane proteins (OMPs). Future work should focus on the development of inhibitors targeting SR modulators, with the aim of creating adjuvants that enhance the efficacy of β-lactam antibiotics.

## MATERIALS AND METHODS

### Bacterial strains and growth conditions

The WT *V. cholerae* strain N16961 and previously constructed (p)ppGpp variant strains of *V. cholerae* and other strains constructed and used in this study are mentioned in Table 1. (p)ppGpp variant strains used were N16961-R13(N16:∆*relA*), N16961-RV1(N16:∆*relV*), BS1.1 (N16:∆*relA* ∆*spoT*), and BRV1(N16:∆*relA* ∆*spoT* ∆*relV*) (11, 12) (N16 is the abbreviation for the wild-type strain N16961). We have constructed DksA mutant strains of N16961 and of (p)ppGpp variant strains. All the plasmids used in this study are mentioned in Table S3. For liquid culture, the strains were grown in Luria broth (LB) at 37°C in a shaker with 180 rpm, whereas LB agar plates were used for solid culture. The following antibiotic concentrations were used: streptomycin (100 µg/mL), spectinomycin (50 µg/mL), kanamycin (40 µg/mL), zeocin (25 µg/mL), ampicillin (100 µg/mL), and chloramphenicol (30 µg/mL for *E. coli* and 2 µg/mL for *V. cholerae*). The bacteria were tested for sucrose sensitivity by plating them onto LA supplemented with 15% sucrose and incubating them at 24°C. For long-term storage at –80°C, we used LB supplemented with 20% glycerol.

### Molecular biological methods

Unless otherwise specified, conventional molecular biology procedures were used for chromosomal and plasmid DNA isolations, electroelution, restriction enzyme digestion, ligation of DNA fragments, bacterial transformation, conjugation, and so on. All restriction enzymes and nucleic acid-modifying enzymes were sourced from New England BioLabs, Inc. and applied in accordance with the manufacturer’s instructions. Transformants were chosen by plating transformed cells on LB agar plates with antibiotics.

### Development of recombinant vectors and mutant strains

Gene deletions and replacements were carried out utilizing the allelic exchange technique using suicide vector pDS132 derivatives. Using specific primer combinations, 500- to 700-base pair (bp) homologous areas upstream and downstream of the corresponding ORF were PCR-amplified (Table S4). The amplified components were purified even before restriction enzyme digestion and ligated into a likewise digested suicide vector. The host bacterium *E. coli* FCV14 was utilized to select and replicate recombinant vectors. The recombinant vectors were transferred to *V. cholerae* via conjugation or electroporation. For conjugation, we used *E. coli* β−2163 as a donor. The mutants were selected on respective antibiotics and further confirmed by PCR. To complement the function of DksA, the complete open reading frame (ORF) of the dksA gene was amplified from N16961 and cloned into the arabinose-inducible integrative vector pBD62 under the control of the P_BAD_ promoter. Cultures were treated with 0.05% arabinose to induce DksA expression.

### Antibiotic susceptibility testing

Antibiotic susceptibility testing was done by disc diffusion method to measure the zone of inhibition and by minimum inhibitory concentration (MIC) determined by broth dilution method. For the disc diffusion method, all the strains were grown overnight aerobically at 37°C in MHB medium and the primary cultures were diluted 1:100 in fresh MHB medium and incubated aerobically at 37°C, when OD_600_ reached 0.5. The 1 mL of this culture was plated onto Mueller-Hinton agar (MHA, Difco, USA) plate (23” × 23” cm) using sterile cotton swabs, and commercially available discs (Liofilchem) containing defined amounts of interested antibiotics were placed on it. Plates were incubated overnight at 37°C in a static incubator, and the zone of clearance was measured with the help of antibiotic zone scale. The antibiotic discs used were erythromycin (15 µg), imipenem (10 µg), norfloxacin (10 µg), ampicillin (10 µg), furazolidone(50 µg), rifampicin (30 µg), penicillin (10 µg), nalidixic acid (30 µg), trimethoprim/sulfamethoxazole (25 µg), gentamicin (10 µg), doxycycline (30 µg), ciprofloxacin (5 µg), neomycin (30 µg), and carbenicillin (100 µg).

Formula used:

The relative zone of inhibition = zone of inhibition of mutant strain/zone of inhibition of N16961 strain.

MIC testing for all (p)ppGpp and DksA mutant strains, along with the WT strain, was done for all 14 antibiotics mentioned above, according to CLSI guidelines. When the OD_600_ of the secondary culture reached 0.5, it was then diluted at 1:1,000 in fresh MHB (∼2 × 10^5^ CFU/mL). Then, 100 µL of this diluted culture was added into each well of a 96-well microtiter polystyrene tray. A series of 2-fold dilutions of an antibiotic was made in this 96-well plate. The mixtures were incubated at 37°C for 16–18 h. MIC was defined as the lowest antibiotic concentration that inhibited visible bacteria growth.

### Growth assay by CFU count method

Growth assay of *V. cholerae* strains N16961, N16:∆*relA*∆*spoT*, N16:∆*relV*∆*relA*∆*spoT*, N16:Δ*dksA*, N16:∆*relA*∆*spoT*Δ*dksA*, N16:∆*relV*∆*relA*∆*spoT*∆*dksA*, N16:∆*relA*∆*dksA*, N16:∆*relV*∆*relA*∆*dksA*, and N16:∆*relV*∆*dksA* after exposure to sub-lethal concentration of antibiotics was measured by determining the number of CFU/ml. Overnight cultures were diluted 100-fold in 10 mL of fresh medium and incubated at 37°C with shaking at 180 rpm/min to reach OD_600_ = 0.5 (∼2 × 10^8^ CFU/mL). Aliquots of 5 mL were then transferred into two different 50 mL tubes. Sub-lethal concentration of antibiotics was added in one tube while another tube having no antibiotics was used as control. Tubes were incubated with shaking at 37°C for 4 h. For the determination of CFU, 1 mL aliquots were removed at the indicated time and cells were harvested by spinning down (8,000 rpm for 2 min) followed by resuspension in fresh medium. Culture was serially diluted and plated on LB agar having an appropriate sub-lethal concentration of antibiotics. Four antibiotics were selected primarily acting on cell wall synthesis for growth assay.

Formula used:

Percentage of Growth = (Growth with antibiotic)/(Growth without antibiotic)×100

Here, growth with antibiotic = total CFU at t = 4 with antibiotic treatment – total CFU at t = 0

Growth without antibiotic = total CFU at t = 4 without antibiotic treatment – total CFU at t = 0

### Sample preparation for whole cell metabolome analysis

For the study of total cellular metabolites, the WT strain N16961 and different (p)ppGpp synthetic and *dksA* gene-deleted strains of *V. cholerae* were grown in LB at 37°C. An overnight culture of 1 mL was pelleted down by centrifugation (10,000 rpm at 4°C for 10 min; Eppendorf centrifuge 5810R) and washed thrice with M9 minimal (M9M) medium. Culture was then diluted 100-fold in fresh casamino acid glucose-M9M medium and grown to late log phase (OD_600_ = 1.0). The cells were pelleted down again by centrifugation (10,000 rpm at 4°C for 10 min), washed with 0.9% normal saline, and stored at −80°C. To extract the intracellular metabolites, cold 100% methanol was added (Sigma Aldrich; Cat no. 34860), followed by vortexing and bath sonication for 10 min (Bransonic Ultrasonic M Cleaning Bath 1510). The cell debris was pelleted down by centrifugation (10,000 rpm at 4°C for 10 min), and the supernatant was collected in two separate microcentrifuge tubes (120 µl each tube), vacuum dried (Thermo Scientific Savant SPD1010), and stored at −80°C. For the analysis of metabolites, the dried supernatant was dissolved in 60 µL of 15% methanol or 50% acetonitrile (Cat no. 271004), followed by vortexing for 5 min and centrifuged (10,000 rpm for 10 min). The supernatant was collected in a separate sample vial (Supelco Analytical). A small amount of each sample was also used to make a pool. Samples were submitted to run in six sets for the mass spectrometry.

### Measurement of metabolites

Data were acquired using an Orbitrap Fusion Mass Spectrometer (Thermo Scientific) in conjunction with a heated electrospray ion source. With minor adjustments, data-gathering procedures were carried out in accordance with published methods (45, 46). In summary, mass resolution was retained at 120,000 for MS1 mode and 30,000 for MS2 acquisition. The data acquisition mass range was 60–900 Da. UPLC ultimate 3,000 was used to separate extracted metabolites. Data were collected using a reverse phase (RP) and HILIC column for positive and negative ionization modes. HSS T3 was used in the RP column, whereas XBridge BEH Amide was used in the HILIC column (Waters Corporation). Solvent A consisted of 20 mM ammonium acetate (pH-9.0) water for polar compound separation, whereas mobile phase B consisted of 100% acetonitrile. At a flow rate of 0.35 mL/min, the elution gradient commences at 85% B and proceeds to 10% B over 14 min. Solvent A for the RP was water, and solvent B was methanol, with 0.1% formic acid in each. At a flow rate of 0.3 mL/min, the elution gradient proceeds from 1% B to 95% B in 10 min. The injection volume of the sample was 5 µL. A pool quality control (QC) sample was taken after every five samples to evaluate signal variation and drift in mass inaccuracy.

### Data processing and analysis

The Progenesis QI software application (Water Corporation) for metabolomics was used to process all LC/MS obtained data using the default settings. The untargeted workflow of Progenesis QI was used to achieve retention time alignment, deconvolution, feature identification, and elemental composition estimations. For database search, the Progenesis QI Metascope plug was used for the in-house library with right mass, retention period, and fragmentation pattern. For added identification certainty, we employed an online spectrum library. The retention time match cut-off in Progenesis QI was 0.5 min, and spectral similarity was more than 30% fragmentation match. Peaks in pool QC samples with a coefficient of variation (CV) of less than 30% were saved for further analysis. Furthermore, each found characteristic was thoroughly confirmed before being used to choose relevant peaks.

For the univariate and multivariate analyses performed with Metaboanalyst 5.0, the resulting data matrices were sum normalized, log-transformed, and Pareto scaled. Principal component analysis (PCA) was done to understand the clustering pattern. Metabolites with a fold change threshold of 2 and above and metabolites passing FDR-adjusted-p-value were considered for the study. Metaboanalyst 5.0 enrichment analysis was used to evaluate the top chemical classes in mutant strains. Heatmap analyses were performed to determine features of statistical significance among mutant groups. Information from EcoCyc for metabolic pathway analysis was used. The list of the metabolites for all the mutant strains analyzed is provided in File S1.

### Whole cell proteome analysis

For the study of total cell proteins, the WT strain N16961 and N16:Δ*relA*Δ*relV*Δ*spoT*, N16:Δ*dksA,* and N16:Δ*relA*Δ*relV*Δ*spoT*Δ*dksA* mutants of *V. cholerae* were selected for proteome analysis. The organisms were grown in LB medium at 37°C with shaking at 180 rpm overnight. Overnight culture of 1 mL was pelleted down and washed thrice with M9M medium. Culture was then diluted 100-fold in fresh casamino acid glucose-M9M medium and grown to late log phase (OD_600_ = 1.0). The cells were pelleted down by centrifugation, washed with chilled PBS, and whole-cell proteins were extracted using lysis buffer (8M urea, 2% sodium deoxycholate in 50 mM ammonium bicarbonate, pH 7.2). Protein samples were digested into peptides using trypsin before reduction and alkylation with dithiothreitol and iodoacetamide, respectively. The resulting peptide mixtures were desalted and analyzed by Eksigent microLC, connected with a TripleTOF 5600 mass spectrometer. In addition, 10 µg desalted peptides was loaded on a reverse phase analytical column (3C18-CL, 300 µm × 15 mm, 3 µm, 120 Å) using mobile phases as follows: water/acetonitrile/formic acid (A, 98/2/0.2%; B, 2/98/0.2%). Separation was established by the following gradient condition: initial 5% B for 5 min, followed by a linear gradient from 3% B to 25% B in 68 min, followed by another linear gradient to 35% B in 73 min. Following the peptide elution window, the gradient was increased to 80% B in 2 min and held for 3 min. Initial chromatographic conditions were restored in 1 min and maintained for 8 min. DDA spectra were acquired using an ESI ion source with a 100–1500 m/z mass range and cycle time 2.3 s. A set of 83 overlapping SWATH windows were also used to acquire the data in DIA mode with a total duty cycle of 4.02 s. Identification of proteins was carried out via a search against Uniprot protein databases of *V. cholerae* with ProteinPilot to obtain a spectral library. The proteins and associated peptides were filtered by PeakView and MarkerView for relative quantitative and qualitative analysis. All the proteins with fold change thresholds of 1.5 and above and *P*-value < 0.05 were considered for the study (File S2).

### Outer membrane permeability analysis

A single colony of WT and mutant strains of *V. cholerae* was inoculated into 5 mL LB broth in a glass culture tube and incubated overnight in a shaking incubator at 180 rpm at 37°C, and 1 mL of overnight culture was pelleted down and washed thrice with M9M medium. The culture was then diluted 100-fold in fresh casamino glucose-M9M medium and grown to late-log phage (OD_600_ = 1.0). The cells were pelleted down at 4°C, and after washing twice with chilled PBS, resuspended in the same volume of PBS. Additionally, 200 µL was added to each 96-well plate. For outer membrane permeability, 1-N-phenylnaphthylamine (NPN) was used at a final concentration of 15 µM. The reading was taken immediately at an excitation wavelength of 350 nm and an emission wavelength of 420 nm. The experiment was done in triplicates.

### Inner membrane permeability assay

For inner membrane permeability, propidium iodide was used at a final concentration of 2.5 µg/mL. The reading was taken at an excitation wavelength of 535 nm and an emission wavelength of 617 nm. The experiment was done in triplicates.

### Real-time RT-PCR analysis of porin expression

To assess the differential expression of porin genes *ompU* and *ompT* in N16961 and N16:Δ*relA*Δ*relV*Δ*spoT*, N16:Δ*dksA,* and N16:Δ*relA*Δ*relV*Δ*spoT*Δ*dksA* mutants of *V. cholerae*, real-time reverse transcriptase PCR was performed. Briefly, the strains were grown to mid-log culture (0.5 OD_600_) in MHB media both in the presence and absence of sub-lethal concentration of antibiotic ampicillin (1 µg/mL). The bacterial cells were then collected by centrifugation, and total RNA was extracted using trizol method. First-strand cDNA was synthesized using Qiagen’s Quantitect Reverse Transcription kit from 1,000 ng RNA. Real-time PCR was carried out in a QuantiStudio 6 Real-time PCR machine of Thermo Fisher Scientific using SYBR Green Master Mix. The primers used are listed in Table S4. RpoB served as indigenous control.

## Data Availability

The SWATH-MS data have been deposited to the ProteomeXchange consortium via the PRIDE partner repository (Project accession: PXD055059) and Metabolomics data deposited to metabolomics workbench (47) with study_ID: ST003711.

## SUPPLEMENTAL MATERIAL

Data S1 spectrum.01169-24-s0001.csv Metabolomics profile of *Vibrio cholerae* strains.

Data S2 spectrum.01169-24-s0002.xlsx Proteomic profiles of *Vibrio cholerae* strains.

Fig. S1 spectrum.01169-24-s0003.pdf Relative zone of inhibition of *V. cholerae* N16961, N16:∆relV , N16:∆relA , N16:∆relV∆relA , N16:∆relV∆dksA, N16:∆relA∆dksA and N16:∆relV∆relA∆dksA strains with different antibiotics.

Fig. S2 spectrum.01169-24-s0004.tiff Volcano plot of A) N16:ΔrelAΔrelVΔspoT, and B) N16:ΔdksA strains of the 291 identified metabolites by LC-MS.

Fig. S3 spectrum.01169-24-s0005.tiff (A) Box plot of DAP decarboxylase protein intensities (mean area under the curve) for N16961, N16:ΔrelAΔrelVΔspoT, N16:ΔdksA and N16:ΔrelAΔrelVΔspoTΔdksA strains and (B) relative fold change in OmpU and OmpT determined by qRT-PCR analysis in mutant strains compared to WT-type strains.

Supplemental material spectrum.01169-24-s0006.pdf Legends for all three supplementary figures.

Supplemental tables spectrum.01169-24-s0007.pdf Tables S1 to S4.

ASM does not own the copyrights to Supplemental Material that may be linked to, or accessed through, an article. The authors have granted ASM a non-exclusive, world-wide license to publish the Supplemental Material files. Please contact the corresponding author directly for reuse.
