# Supplementary figures and images for: (p)ppGpp and DksA play a crucial role in reducing the efficacy of β-lactam antibiotics by modulating bacterial membrane permeability
Source: Microbiol Spectr. 2025 Feb 24;13(4):e01169-24. doi: 10.1128/spectrum.01169-24 (PMC11960062; doi:10.1128/spectrum.01169-24)

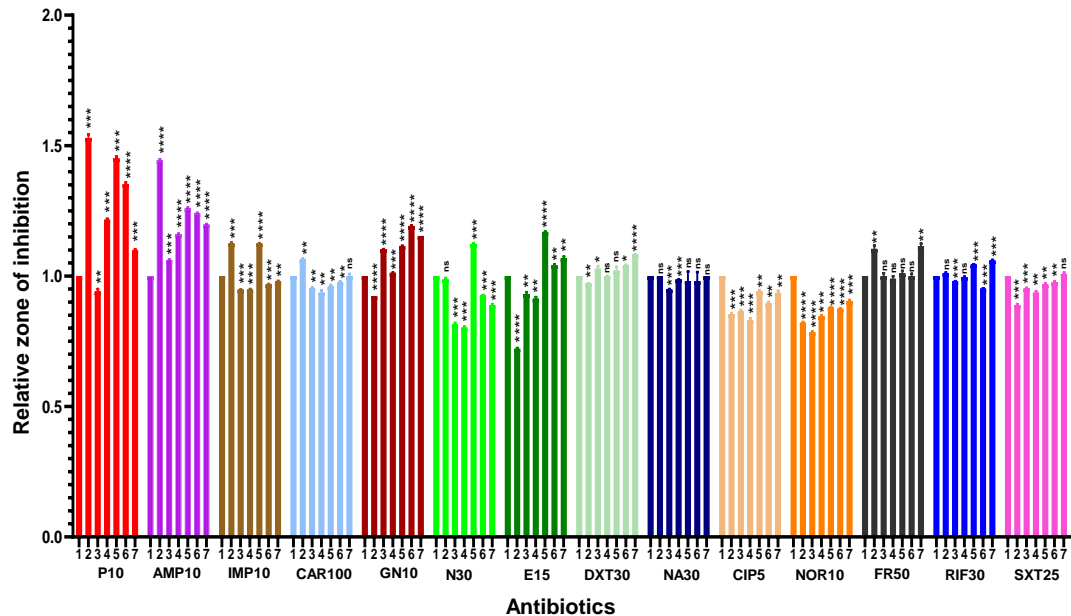

Supplement: Fig. S1 — Relative zone of inhibition of V. cholerae N16961, N16:∆relV , N16:∆relA , N16:∆relV∆relA , N16:∆relV∆dksA, N16:∆relA∆dksA and N16:∆relV∆relA∆dksA strains with different antibiotics. [file spectrum.01169-24-s0003.pdf]

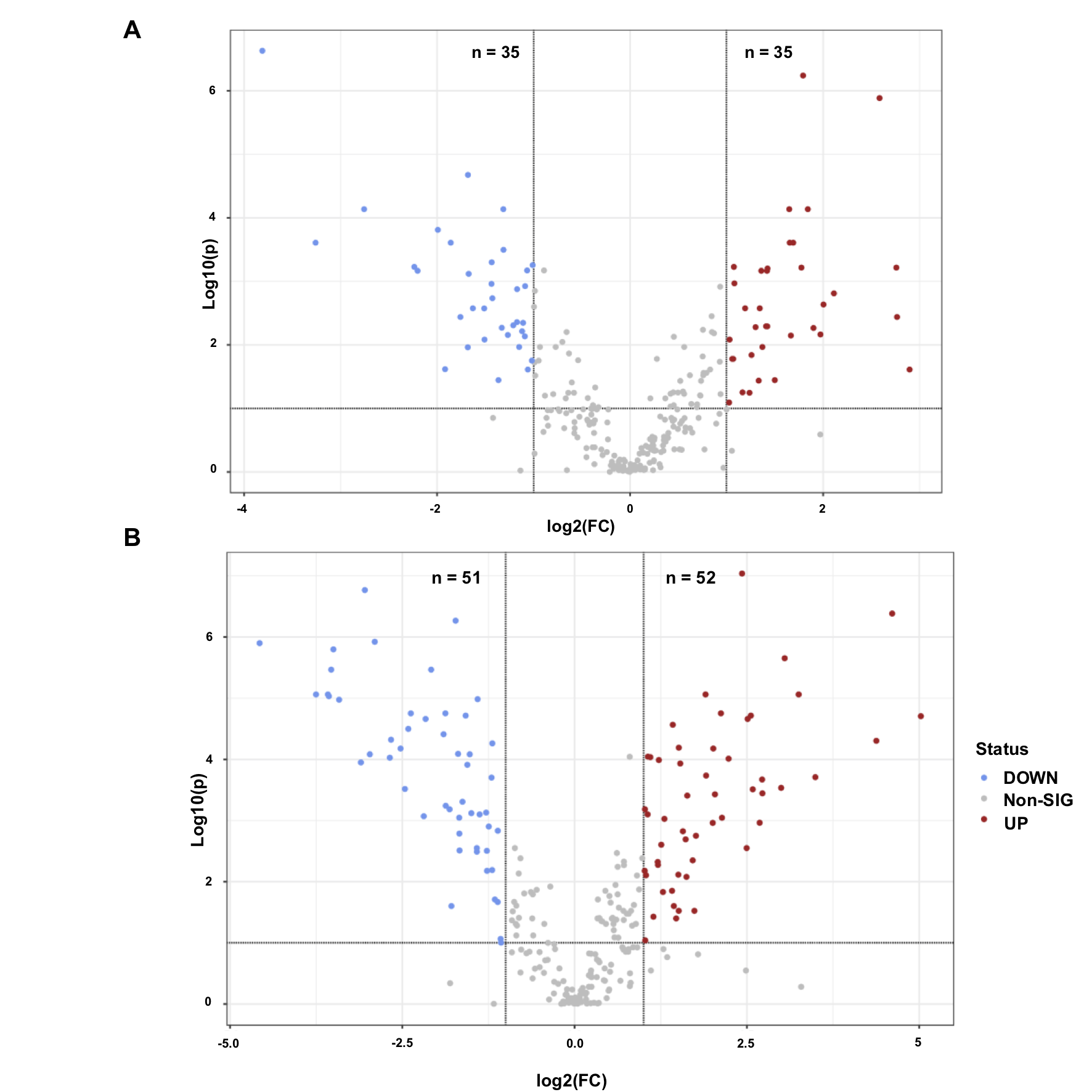

Supplement: Fig. S2 — Volcano plot of A) N16:ΔrelAΔrelVΔspoT, and B) N16:ΔdksA strains of the 291 identified metabolites by LC-MS. [file spectrum.01169-24-s0004.tiff]

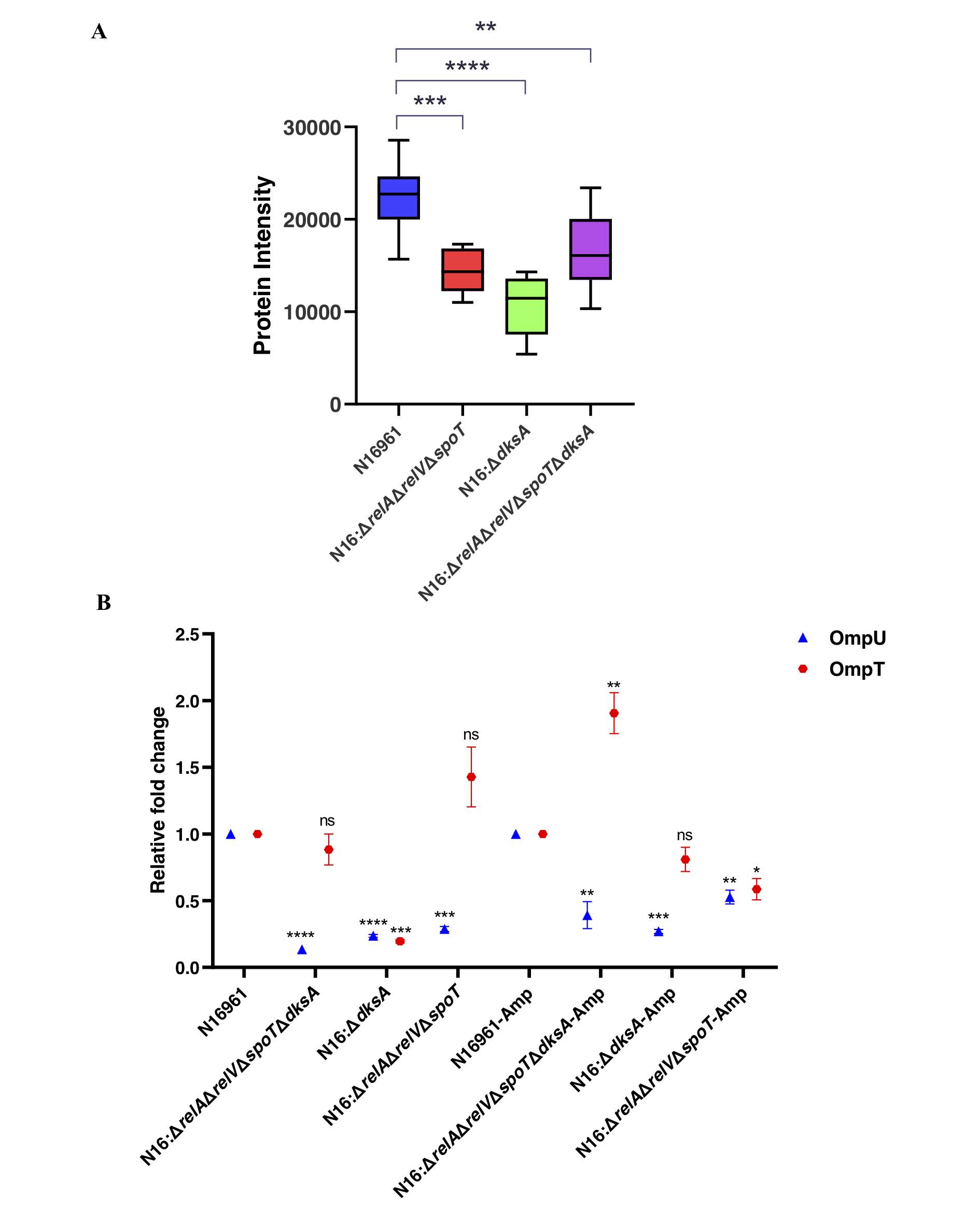

Supplement: Fig. S3 — (A) Box plot of DAP decarboxylase protein intensities (mean area under the curve) for N16961, N16:ΔrelAΔrelVΔspoT, N16:ΔdksA and N16:ΔrelAΔrelVΔspoTΔdksA strains and (B) relative fold change in OmpU and OmpT determined by qRT-PCR analysis in mutant strains compared to WT-type strains. [file spectrum.01169-24-s0005.tiff]
