# Supplementary material for: (p)ppGpp and DksA play a crucial role in reducing the efficacy of β-lactam antibiotics by modulating bacterial membrane permeability
Source: Microbiol Spectr. 2025 Feb 24;13(4):e01169-24. doi: 10.1128/spectrum.01169-24 (PMC11960062; doi:10.1128/spectrum.01169-24)
Supplement: Supplemental material — Legends for all three supplementary figures. [file spectrum.01169-24-s0006.pdf]

Legend to Supplementary Figures

Supplementary Figure 1: The graph represents the relative zone of inhibition of *V. cholerae* N16961, N16:Δ*relV* , N16:Δ*relA* , N16:Δ*relV*Δ*relA* , N16:Δ*relV*Δ*dksA* N16:Δ*relA*Δ*dksA* and N16:Δ*relV*Δ*relA*Δ*dksA* strains with different antibiotics.

Supplementary Figure 2: Volcano plot of **A)** N16:Δ*relA*Δ*relV*Δ*spoT*, and **B)** N16:Δ*dksA* strains of the 291 identified metabolites by LC–MS. The volcano plot shows the fold-change (x-axis) versus the significance (y-axis) of the 291 metabolites. The vertical and horizontal dotted lines show the cut-off of fold-change = ± 2, and of p-value = 0.05, respectively.

Supplementary Figure 3: **A)** Box plot of DAP decarboxylase protein intensities (mean area under the curve) for N16961, N16:Δ*relA*Δ*relV*Δ*spoT*, N16:Δ*dksA* and N16:Δ*relA*Δ*relV*Δ*spoT*Δ*dksA* strains and **B)** Relative fold change in OmpU and OmpT determined by quantitative real-time PCR (qRT-PCR) analysis in mutant compared to WT-type strains. All data were normalized with RpoB and given as relative to WT-strain. Statistical significance was calculated using Welch's t-test, ($p < 0.05$).
