# Supplementary material for: (p)ppGpp and DksA play a crucial role in reducing the efficacy of β-lactam antibiotics by modulating bacterial membrane permeability
Source: Microbiol Spectr. 2025 Feb 24;13(4):e01169-24. doi: 10.1128/spectrum.01169-24 (PMC11960062; doi:10.1128/spectrum.01169-24)
Supplement: Supplemental tables — Tables S1 to S4. [file spectrum.01169-24-s0007.pdf]

Table S1: Minimum Inhibitory concentration (MIC) of fourteen different antibiotics for mutant strains.

Antibiotics	N16961	N16:Δ*relV*	N16:Δ*relV*Δ*dksA*	N16:Δ*relA*	N16:Δ*relA* Δ*dksA*	N16:Δ*relA* Δ*relV*	N16:Δ*relA* Δ*relV*Δ*dksA*
**Penicillin**	5 µg/ml	5 µg/ml	2.5 µg/ml	5 µg/ml	2.5 µg/ml	5 µg/ml	2.5 µg/ml
**Ampicillin**	20 µg/ml	20 µg/ml	10 µg/ml	20 µg/ml	10 µg/ml	20 µg/ml	10 µg/ml
**Imipenem**	5 µg/ml	5 µg/ml	5 µg/ml	5 µg/ml	5 µg/ml	5 µg/ml	5 µg/ml
**Carbenicillin**	2.5 µg/ml	2.5 µg/ml	2.5 µg/ml	2.5 µg/ml	2.5 µg/ml	2.5 µg/ml	2.5 µg/ml
**Gentamicin**	5 µg/ml	5 µg/ml	5 µg/ml	5 µg/ml	5 µg/ml	5 µg/ml	5 µg/ml
**Neomycin**	20 µg/ml	20 µg/ml	20 µg/ml	>40 µg/ml	>40 µg/ml	>40 µg/ml	>40 µg/ml
**Erythromycin**	2.5 µg/ml	2.5 µg/ml	1.25 µg/ml	2.5 µg/ml	1.25 µg/ml	2.5 µg/ml	1.25 µg/ml
**Doxycycline**	100 ng/ml	100 ng/ml	100 ng/ml	100 ng/ml	100 ng/ml	100 ng/ml	100 ng/ml
**Nalidixic acid**	0.3125 µg/ml	0.3125 µg/ml	0.3125 µg/ml	0.3125 µg/ml	0.3125 µg/ml	0.3125 µg/ml	0.3125 µg/ml
**Ciprofloxacin**	6.25 ng/ml	12.5 ng/ml	6.25 ng/ml	12.5 ng/ml	6.25 ng/ml	12.5 ng/ml	6.25 ng/ml
**Norfloxacin**	15.63 ng/ml	15.63 ng/ml	15.63 ng/ml	15.63 ng/ml	15.63 ng/ml	15.63 ng/ml	15.63 ng/ml
**Furazolidone**	0.625 µg/ml	0.625 µg/ml	0.625 µg/ml	0.625 µg/ml	0.625 µg/ml	0.625 µg/ml	0.625 µg/ml
**Rifampicin**	50 ng/ml	50 ng/ml	25 ng/ml	50 ng/ml	25 ng/ml	50 ng/ml	25 ng/ml
**Sulfamethoxazole**	20 µg/ml	40 µg/ml	10 µg/ml	40 µg/ml	20 µg/ml	40 µg/ml	20 µg/ml

Table S2: Minimum Inhibitory concentration of six antibiotics for DksA complemented strains.

Antibiotics	N16961	N16:Δ*relA*Δ*relV*Δ*spoT*Δ *dksA*::pBD62-DksA	N16:Δ*dksA*::pBD62 -DksA	N16:Δ*dksA*::pBD62	N16:Δ*relA*Δ*relV*Δ*sp oT*Δ*dksA*::pBD62
**Penicillin**	5 µg/ml	>20 µg/ml	>20 µg/ml	2.5 µg/ml	2.5 µg/ml
**Ampicillin**	20 µg/ml	>20 µg/ml	>20 µg/ml	10 µg/ml	10 µg/ml
**Erythromycin**	2.5 µg/ml	2.5 µg/ml	2.5 µg/ml	1.25 µg/ml	1.25 µg/ml
**Doxycycline**	100 ng/ml	100 ng/ml	100 ng/ml	100 ng/ml	25 ng/ml
**Rifampicin**	50 ng/ml	25 ng/ml	50 ng/ml	25 ng/ml	12.5 ng/ml
**Sulfamethoxazole**	20 µg/ml	5 µg/ml	5 µg/ml	5 µg/ml	5 µg/ml

Table S3: Plasmids used in the study

Plasmids	Genotype and phenotype	References
pDS132	*oriR6K mobRP4 sacB cat*; conjugative vector, Cam^r	Laboratory stock
pEM7	*pUC ori bla sh ble;* Ampr *,* Zeo^r	Laboratory stock
pBD62	pSW23T *sh ble* oriR6K, mobRP4, attPctx, Zeo^r	Das *et al.,* 2014
pJV08	pDS132:Up *dksA:kan* -Dwn *dksA*; Kan^r, Cam^r	This study
pJM1	pDS132: Up *dksA-sh ble* -Dwn *dksA*; Cam^r, Zeo^r	This study
pTS09	*dksA* complementation vector (pBD62:dksA), Zeo^r	This study
pMC1	*dksA* complementation vector (pTS09:Δ*sh ble*::*bla*), Amp^r	This study

Table S4: Primers used in the study

Primer name	Sequence (5'-3')	RE site	Target
693(*dksA*-F)	GCGAGCTCGCGTGATTCTGTTTCGAGAG	SacI	Amplification of *dksA* Up and Down cassette from *V. cholerae*
694(*dksA*-R)	CCTCTAGAGGTTCGGTGATCACCATGC	XbaI	
1365 (*shble-F)*	CGCTGCAGGCGATATCGCTAGCTCGAGC	PstI	Amplification of *sh ble* from pEM7
1366 *(shble-R)*	CGCTGCAGCGGGAATTCTCAGTCCTGC	PstI	
1262 F(dksA)	GCACTAGTACCATGACAGAGTCTAAAAAGAA	Spe1	*dksA* complementation
1263 R(dksA)	CGGAGCTCTTAGCCAAGCATTTGCTTTTC	SacI	
727F	CCATGGATGTATCCGCTCATGAGAC	NcoI	
1399R	GCTCGCGAAACTTGGTCTGACAGTTACC	NruI	
1709 (OmpU-F)	TTGACTACACTGGCTAC	-	Real time PCR of OmpU
1710 (OmpU-R)	GTAAGAGCGGAAGTTTG	-	
1711 (OmpT-F)	GCACTGATTGGTGTTAC	-	Real time PCR of OmpT
1712 (OmpT-R)	CTCATAGCCAGCATACA	-	
968 (RpoB-F)	CTGTCTCAAGCCGGTTACAA	-	Real time PCR of RpoB
969 (RpoB-R)	ATGCGATCTGTCGTGCTATC	-	
